# Poor dietary practice and associated factors among type-2 diabetes mellitus patients on follow-up in Nigist Eleni Mohammed Memorial Teaching Hospital, Ethiopia

**DOI:** 10.11604/pamj.2022.41.164.28675

**Published:** 2022-02-23

**Authors:** Mulugeta Selassie Erkocho, Dessalegn Tamiru Adugna, Tegegn Tadesse Arficho, Adisalem Gizachew Azene

**Affiliations:** 1 Hadiya Zone Health Department, Hossana, Ethiopia,; 2 Nutrition and Dietetics Department, Public Health Faculty, Jimma University, Jimma, Ethiopia,; 3 Wachamo University, Department of Public Health, Hossana, Ethiopia

**Keywords:** Practice, type-2 diabetes, diet, Ethiopia

## Abstract

**Introduction:**

good dietary practice is one of the top pillars of self-care among patients of diabetes mellitus. However, the dietary practice of patients attending health institutions in the study area was not studied. Therefore, the prevalence and associated factors of poor dietary practice were determined among diabetic patients on follow-up in Nigist Eleni Mohammed Memorial Referral and Teaching Hospital, Southern Ethiopia.

**Methods:**

facility-based cross-sectional study design was employed on type-2 diabetes mellitus adult patients. The study was conducted from March to April 2020. Total sample size was 322. Systematic random sampling method was used to select the study respondents. Odds ratio and their 95% confidence intervals together with P-value ≤0.05 were used to identify independent predictors of poor dietary practice during multivariable logistic regression.

**Results:**

the prevalence of the poor dietary practice among type diabetes patients was 53.7% (n=168). Low wealth status AOR 3.34, 95% CI: 1.50-7.41; p-value=0.003 and absence of family and friends support AOR 4.80, 95% CI: 2.54-9.0 and P-value<0.001 were associated factors with poor dietary practice among type-2 diabetes patients.

**Conclusion:**

the overall prevalence of poor dietary practice among type-2 diabetic adult patients was high that not going in line with international recommendations on dietary management of the diabetes mellitus. Integrated governmental and non-governmental activities should be in place to improve the economic status of type-2 diabetic patients. Support from the family members is found to be essential factor to promote dietary practice among type-2 diabetic adult patients.

## Introduction

Diabetes mellitus (DM) is a clinical syndrome characterized by hyperglycemia due to absolute or relative deficiency of insulin. Diabetes mellitus is clinically categorized as type I diabetes, type II diabetes, gestational diabetes mellitus and other specific types of diabetes due to other causes such as genetic defects in b-cell function, genetic defects in insulin action and diseases of the exocrine pancreas [1]. The international diabetes federation estimates that 463 million adults are living with diabetes worldwide that 1 in 11 people living with diabetes. Ongoing patient self-management, education, and support are critical to prevent acute complications and to reduce the risk of long-term complications from the disease. It is known that poor dietary practice would lead to life-threatening complications in individuals with diabetes [2]. Ethiopia is among sub-Saharan African countries with a rapidly increasing burden of diabetes mellitus which starts from 1.9 percent in southern Ethiopia to 6.8 percent Northeast Ethiopia [3-5]. Recommended dietary practices or dietary adherence include consuming more fruits and vegetables, nuts and whole grains, and choosing unsaturated vegetable oil as opposed to saturated animal based fats. It also includes limiting consumption of snacks that are high in fat, sugar, or salt [6].

Even though dietary modification is one of the corner stone in type-2 DM management and is usually recommended as the first step, it is considered as one of the most challenging aspects of diabetes management. Implementation of recommended dietary practice for individuals with T2 DM requires collaboration between the patient and the healthcare provider [1]. None adherence to recommended dietary recommendation in diabetes mellitus patients may result in long term complications [7]. Some of the factors associated with poor dietary practice are socio-economic status, duration of illness, duration of follow-up, educational level, coo morbidity, family support, lack of diabetes knowledge, cost of healthy diet and poor communication with healthcare providers [8].

In Ethiopia, studies done in Addis Ababa and northern part of Ethiopia indicated that more than half of the patients had poor practice of diet [9,10]. However, there was no prior study done on dietary practice among diabetes patients in southern part of Ethiopia. Therefore, undertaking evidence-based researches would have a positive impact on designing and implementation of dietary practice programs for people with T2 DM in Ethiopia. Therefore, this study was aimed to determine the prevalence of poor dietary practice and its associated factors among type-2 diabetic patients on follow-up in Nigist Eleni Mohammed Memorial Teaching Hospital, Southern Ethiopia.

## Methods

**Study setting:** the study was conducted Nigist Eleni Mohammed Memorial Referral and Teaching Hospital (NEMMTH) which is located in Hadiya zone in South Nation´s Nationalities and People's Region found in 232 kms south of Addis Ababa, Ethiopia. It provides preventive, curative and rehabilitative clinical services organized in four case teams as outpatient, inpatient, emergency and critical care, maternal, child health and obstetrics and operation theatre. The out patients’ services are given in Outpatient department (OPD) clinics (internal medicine, surgery, pediatrics and child health, gynecologic), specialty clinics (psychiatry, dermatology, ophthalmology, dentistry, orthopedic, referral and consultation clinics, maternal and child health care follow-up clinics). Diabetes mellitus follow-up clinic is under outpatient department which serves cases with diabetes mellitus on regular follow-up. The inpatients services are given under four major departments like internal medicine, general surgery, pediatrics and gynecologic and obstetrics, orthopedics and operation theatre. It has a capacity of accommodating 250 beds [11]. This study was carried out in Nigest Eleni Mohammed Memorial Teaching Hospital, Southern Ethiopia, from March to April 2020 Gregorian calendar (G.C).

**Study design:** facility-based cross-sectional study was employed to assess the prevalence of poor dietary practice among type II diabetes mellitus adult patients. It is because the main objective of the study was on assessing the prevalence and factors associated with the dependent variable.

**Study population:** the study population was all patients with type-2 diabetes aged 18 years and above who were on diabetic follow-up at Nigist Eleni Mohammed Memorial Teaching and Referral Hospital and randomly selected for study. Patients who were diagnosed with type-2 diabetes and on clinical care for at least one year were included. However, those who critically ill and need immediate treatment during data collection period were excluded from the study. By considering non-response rate of 10% the total sample size was 322. Systematic random sampling method was used to select the study subjects. The hospital had a total of 1177 DM patients who were on follow-up and among these patients 862 patients were type-2 DM patients. Each patient visits the facility on monthly basis. The K-value (the interval) was calculated by dividing total number of type-2 diabetic patients (862) from diabetes referral clinic registration log book to the calculated sample size (322). Accordingly, every 3^rd^ patient was selected, with the first sample chosen randomly between 1 and k.

**Data collection instruments and personnel:** data was collected using a structured interviewer administered questionnaire. The questionnaire was initially prepared in English then translated in to local language (Amharic) by professionals who were fluent two languages and then translated back to English to ensure consistency. In order to ensure reliability and consistency, enumerators were trained for 1 day. Pretest was undertaken by considering 5% of the sample size in Homacho hospital located outside the study area. To determine the poor dietary practice of individuals with DM, we used a modified form of the eight items Morisky medication adherence scale (MMAS-8) which was modified by Worku A *et al*. [5]. This scale has 11 components and was computed by taking the mean value to classify the respondents' poor and good dietary practice respectively. Each of the items contain two response options (Yes = 1 and No= 0, here yes was used for those responses which are negatively answered or far true answer from what science is talking about). The questionnaire was tested for internal consistency (reliability) with Cronbach's Alpha test (0.7).

### Operational definitions

**Poor dietary practice:** respondents who answered incorrectly on 10 items of perceived dietary practice questions and those who scored above the mean value were classified as poor dietary practice [12].

**Good dietary adherence:** respondents who answered correctly on 10 items of perceived dietary adherence questions and those who scored equal or below the mean value were classified as good dietary practice [12].

**Good dietary knowledge:** respondents who answered correctly to knowledge related questions and those who scored greater than the overall mean value.

**Poor dietary knowledge:** respondents who answered in-correctly to knowledge related questions and those who scored less than or equal to overall mean value.

### Study variables

**Dependent variable:** adherence to dietary recommendation.

**Independent variable**

**Socio demographic characteristics:** age, sex, religion, marital status, type of work, income, educational status.

**Disease related:** duration of illness, duration of follow-up, other co-morbidity, family support, attending dietary education.

**Food related:** cost of the food, availability of the food, type of food that should be consumed, frequency and meal timings, member of diabetic association.

**Knowledge:** about the recommended diet.

**Data processing and analysis:** data were entered and analyzed by using SPSS version-20 statistical package. House hold wealth index was determined from asset data using principal component analysis (PCA). First, variables were coded between 0 and 1, and then the variables entered and analyzed using PCA and those variables which have commonality values greater than 0.5 were used to produce factor scores. Frequency distributions, percentages, tables and charts were used to show results of univariate analysis. Variables with p-value≤0.25 in bivariate regression were considered as candidates for multivariable logistic regression. Odds ratios and 95% CI together with p-value≤0.05 were used to identify independent factors associated with poor dietary practice. Goodness of fitness of the final model was checked using Hosmer and Lemeshow adequacy of model test.

**Ethical consideration:** ethical clearance was obtained from ethical review committee of Wachemo University School of post-graduate studies [ref no. WCU/SGS/1/73/20]. Official letter of permissions was obtained from Nigist Eleni Mohammed Memorial teaching hospital medical director office. Then verbal consent was obtained from each respondent after telling the purpose of the study. Information was recorded anonymously and confidentiality and beneficence were assured throughout the study.

## Results

**Socio demographic characteristics of the respondents:** from the total 322 eligible respondents 313(97.2%) were participated in the study. More than half of the participants (56.9%) were males. The mean age (±S. D) of type-2 DM patients was 48.12 (±11.348) years with the age ranges between 20-90 years. The majority of the participants 187 (59.7%) were between 41 and 60 years. Among the study subjects, majority of them 214 and 240 (68.4% and 76.7%) were from protestant Christian followers and from Hadiya ethnic group respectively. Regarding educational status, 51 (16.3%) and 99 (31.6%) of the patients attended higher education and had no formal education respectively (Table 1).

**Table 1:** socio-demographic characteristics of type 2 diabetic patients at Nigist Eleni Mohammed Memorial Referral and Teaching Hospital, Hossana, SNNPR, Ethiopia, 2020 (n=313)

Variable	Categories	Frequency (n)	Percentage
**Sex**	Female	135	43.1
	Male	178	56.9
	Less than 40 years	87	27.8
**Age in years**	41-60 years	187	59.7
	>60 years	39	12.5
**Family size**	Less than 3	49	15.7
	4-6	148	47.3
**Religion**	Greater than 6	116	37
	Protestant	214	68.4
	Orthodox	57	18.2
	Muslim	30	9.6
**Marital status**	Catholic	11	3.5
	Married	297	94.9
	Single	16	5.1
**Residence**	Urban	197	62.9
	Rural	116	37.1
	Hadiya	240	76.7
**Ethnicity**	Kembata	25	8
	Gurage	13	4.2
	Amahara	22	7
	Silitie	13	4.2
	Unable to read and write	99	31.6
**Educational status**	Able to read and write	48	15.3
	Primary school	51	16.3
	Secondary school	64	20.4
**Occupation**	Higher education	51	16.3
	Farmer	69	22
	Government employee	83	26.5
	Merchant	71	22.7
	Private organization employee	6	1.9
	Daily laborer	5	1.6
	House wife	73	23.3
**Wealth**	Other	6	2
	Low	109	34.8
	Medium	104	33.2
	High	100	32

**Health related characteristics of the respondents:** the mean duration of patient’s follow-up was 6.12 (SD±4.575) years, which is ranging from 1 to 5 years (51.1%) to greater than 10 years (11.8%). More than one third of participants (35.8%) had chronic disease in addition to type-2 diabetes, mainly hypertension 83 (26.5%) followed by heart disease 15 (4.8%). From the total study participants more than half of the study participants 171 (54.6%) had poor dietary knowledge. Among those who had poor dietary knowledge male accounts 30.6%. Majority of the study participants (82.1%) responded fruit and vegetables as food groups which they used to control their blood glucose level.

**Proportion of poor dietary practice among type-2 diabetic patients:** the overall proportion of poor dietary adherence among type diabetes patients was 168(53.7%) [95% CI (48.2, 58.8)]. According to this study the proportion of poor dietary adherence practice among age groups of less than 40 years was 28.6% [95%CI, (22, 32.6)] and it was 10.7% [95%CI, (8.9, 16.3)] among the age groups of 60 years and above. More than half of the respondents (57.8%) reported that they were an unable to follow dietary recommendation due to unavailability of food items, cost of foods, lack of supports from their families and friends and lack of information. A significant number of the study participants (45%) didn't continue with dietary plan when they felt that their DM is under control. More than 3 in 4 patients (76%) failed to cut down fat intake from their daily foods.

**Factors associated with poor dietary adherence among type II diabetes mellitus adult patients:** family size, residence, educational status, occupation, wealth status, knowledge status of the respondents, duration of follow-up, co-morbidity, fasting blood sugar level, physical exercise, being member of diabetic association, missed dietary planning, cost of foods, access to fruits and vegetables and family and friends support were selected as candidate for multivariable logistic regression. However in multivariable analysis low wealth status AOR 3.34, 95% CI: 1.50-7.41; p-value=0.003 and absence of family and friends support AOR 4.80, 95% CI: 2.54-9.0 and p-value<0.001 were independently associated with poor dietary adherence among type II DM adult patients (Table 2).

**Table 2:** factors associated with dietary adherence in type-2 diabetic patients on follow-up at Nigist Eleni Mohammed Memorial Referral and Teaching Hospital, Hossana, SNNPR, Ethiopia, 2020 (n=313)

Variable	Poor	Good	COR [95%CI]	P-value	AOR [95%CI]	P-value
**Educational status**						
Unable to read and write	62	37	2.209 (1.111-4.393)	0.024	1.165 ( 0.453-2.991)	0.872
Able to read and write	31	17	2.404 (1.069-5.406)	0.034	1.306 (0.487 -3.506 )	0.725
Primary school	30	21	1.883 (0.858-4.133)	0.115	1.003 (0.395 -2.550)	0.969
Secondary school	23	41	0.739 (0.348-1.571)	0.432	0.544 (0.231-1.281	0.283
Higher education	22	29	1		1	
**Wealth**						
Low	68	41	1		1	
Medium	64	40	2.949 ( 1.679- 5.177)	0.000	0.395 (0.197-0.789)	0.009
High	36	64	2.844 ( 1.612- 5.020)	0.001	0.283 (0.14-0.55)	0.001
**Residence**						
Rural	73	43	1.83 (1.140-2.914)	0.012	0.39 (0.18-0.85)	0.18
Urban	95	102	1		1	
**Physical exercise**						
3 times a week for 30	14	28	0.349 ( 0.169-0.721)	0.004	2.244 (0.914 -5.51)	0.078
minutes						
Less than 3 times a week	71	59	0.841 ( 0.520-1.361)	0.481	0.199 (0.666-0.358)	1.239
for 30 minutes						
No physical activity	83	58	1		1	
**Access of fruits and vegetables**						
Yes	137	96	2.256 (1.341 -3.794)	0.002	0.947 (0.46-1.899)	0.883
No	31	49	1		1	
**High cost of foods**						
Yes	138	95	2.421 (1.436 -4.083)	0.001	0.648 (0.263-1.597)	0.346
No	30	50	1		1	
**Family support**						
Yes	129	60	1	0.001	1	<0.001
No	39	85	4.686 (2.878-7.629)		4.80 (2.54-9.0)	

COR: crude odds ratio: AOR: adjusted odds ratio; CI: confidence interval

## Discussion

The overall magnitude of poor dietary adherence to dietary recommendation was 168(53.7%) among diabetic patients on follow-up at Nigist Eleni Mohammed Memorial Referral and Teaching Hospital. Lack of family and friends support and being low in wealth status were the factors found to be associated with poor dietary practice among type-2 diabetes mellitus patients.

According to finding, the overall magnitude of poor dietary practice among type-2 diabetes patients was in line with the research done in Addis Ababa, Ethiopia, which showed that 51.4% of type-2 diabetes patients had poor dietary practice and in Debre Birhan Teaching Hospital that showed 55.7% of the respondents had poor dietary practice among patients [5,8]. Similar findings were reported in a cross-sectional study done by the University of Calgary indicated that 50% of the respondents had poor adherence to the recommended diet [13].

In contrary, findings of this study was higher than the study conducted in Kenya, Mekelle, Felege Hiwot Hospital and Harari (Ethiopia) where 69%, 64.1%, 60% and 59% of participants did not follow dietary recommendations respectively [12,14-16]. The disparity of results among different study area could be due to variation in study setting, adherence measurement tool variation and difference in socio economic status [17]. The finding of this result was lower than the study done in Urban Area of Urmia, Northwest of Iran where 26.2% of participants had poor dietary adherence among patients [18].

Diabetic patients with low wealth status were more likely to have poor dietary practice than those from households having medium wealth status. Respondents who had medium wealth status were 60% less likely to have poor dietary practice than those who had low wealth status. This result is in agreement with a study done in Diredawa Referral Hospital Ethiopia which showed the study participants who had a medium monthly income were less likely to have poor adherence than those who have low wealth status [19]. It is also in line with a report from studies done in Addis Ababa, Tikur Anbessa Specialized Hospital in 2012, Felege Hiwot Hospital, North West Ethiopia, Mekelle Hospital and Ayder Referral Hospitals, Northern Ethiopia [20-22]. This is because of those who have economic constraints have no enough money to buy different types of foods to fulfill their daily requirements. Therefore, they will be forced to consume only some specific foods without choice and get exposed to poor self-management according to diet. Besides an increase in cost of foods might have a negative impact on patients who were from low socio-economic levels in Ethiopia [23,24].

Diabetic patients who lack family and friends support were more likely to be poor in dietary practice than those who have family and friend support. Those patients who lack family and friend support had 4.8 times more odds of having poor dietary practice than their counterparts. Similar results were reported from Addis Ababa [22] and Jimma Medical College, South West Ethiopia [25] which showed a positive association between social support and adherence to lifestyle recommendations among patients. This might be due to the fact that when they couldn't get help from family and friends, they might feel despondent and being prone for missing their dietary meal plan. When people living with diabetes and didn't have support from their close social networks, they might feel stressed to the life, often generating feelings of isolation, frustration, anger and guilt that might damage their healthy food consumption. Social support increases adherence by facilitating self-care activities such as buying foods from groceries and providing motivation to cope with dietary recommendations. This is in agreement with the study finding in Bahrain [26]. Similarly, this finding also agrees with the study findings in a cross-sectional study done in Taiwan and Nekemte Referral Hospital, Ethiopia. The studies show that family and social support have a positive impact on recommended dietary adherence practice among diabetic patients [27].

The strength of this study might be the use of standardized questionnaire that was prepared for assessing an adherence for recommended dietary practices among diabetic patients. Assessing the level of dietary practice using self-reported dietary practice has limitations that might expose for recall bias or social desirability bias. This study finding may not show temporal relationships of potential risk factors with dietary adherence due to cross-sectional nature of the study design used.

## Conclusion

The study indicates that poor dietary practice among type-2 diabetic patients is higher that needs strengthening nutrition education and activities related with nutrition for patients. Diabetic mellitus patients who lack of family and friends support are more prone for poor dietary practice and those having low household wealth status also are more likely to have poor dietary practice which obstacles the care of patients on follow-up for diabetes mellitus. Integrated Governmental and nongovernmental activities should be in place to improve the economic status of type-2 diabetic patients. Lastly, supports from the family members and friends are found to be essential factor to promote dietary practice among type-2 diabetic adult patients.

### What is known about this topic

- It is known that poor dietary practice would lead to life-threatening complications in individuals with diabetes;
- Socioeconomic status, duration of illness, duration of follow-up, educational level, co-morbidity, family support, lack of diabetes knowledge, cost of healthy diet and poor communication with healthcare providers were factors associated with poor dietary practice among type-2 diabetes mellitus patients;
- Ongoing patient self-management, education, and support are critical to prevent acute complications and to reduce the risk of long-term complications from the disease.

### What this study adds

- Identified the context based prevalence of poor dietary practice among type-2 patients on follow-up in health facility;
- The study might fill the information gap depending on dietary self-care among cases of diabetes mellitus;
- Not being the member of the diabetic association was a factor found to be associated with poor dietary practice among patients with type-2 DM.
